# Human Wharton’s Jelly Mesenchymal Stem Cells Plasticity Augments Scar-Free Skin Wound Healing with Hair Growth

**DOI:** 10.1371/journal.pone.0093726

**Published:** 2014-04-15

**Authors:** Vikram Sabapathy, Balasubramanian Sundaram, Sreelakshmi VM, Pratheesh Mankuzhy, Sanjay Kumar

**Affiliations:** 1 Center for Stem Cell Research (CSCR), Christian Medical College (CMC), Bagayam, Vellore, Tamil Nadu, India; 2 School of Bioscience and Technology, VIT University, Vellore, Tamil Nadu, India; Indian Institute of Toxicology Reserach, India

## Abstract

Human mesenchymal stem cells (MSCs) are a promising candidate for cell-based transplantation and regenerative medicine therapies. Thus in the present study Wharton’s Jelly Mesenchymal Stem Cells (WJ-MSCs) have been derived from extra embryonic umbilical cord matrix following removal of both arteries and vein. Also, to overcome the clinical limitations posed by fetal bovine serum (FBS) supplementation because of xenogeneic origin of FBS, usual FBS cell culture supplement has been replaced with human platelet lysate (HPL). Apart from general characteristic features of bone marrow-derived MSCs, wharton jelly-derived MSCs have the ability to maintain phenotypic attributes, cell growth kinetics, cell cycle pattern, *in vitro* multilineage differentiation plasticity, apoptotic pattern, normal karyotype-like intrinsic mesenchymal stem cell properties in long-term *in vitro* cultures. Moreover, the WJ-MSCs exhibited the *in vitro* multilineage differentiation capacity by giving rise to differentiated cells of not only mesodermal lineage but also to the cells of ectodermal and endodermal lineage. Also, WJ-MSC did not present any aberrant cell state upon *in vivo* transplantation in SCID mice and *in vitro* soft agar assays. The immunomodulatory potential assessed by gene expression levels of immunomodulatory factors upon exposure to inflammatory cytokines in the fetal WJ-MSCs was relatively higher compared to adult bone marrow-derived MSCs. WJ-MSCs seeded on decellularized amniotic membrane scaffold transplantation on the skin injury of SCID mice model demonstrates that combination of WJ-MSCs and decellularized amniotic membrane scaffold exhibited significantly better wound-healing capabilities, having reduced scar formation with hair growth and improved biomechanical properties of regenerated skin compared to WJ-MSCs alone. Further, our experimental data indicate that indocyanin green (ICG) at optimal concentration can be resourcefully used for labeling of stem cells and *in vivo* tracking by near infrared fluorescence non-invasive live cell imaging of labelled transplanted cells, thus proving its utility for therapeutic applications.

## Introduction

Mesenchymal stromal cells (MSCs) are a pluripotent class of stem cells that has the ability to self-renew and differentiate into multiple cell lineages. Friedenstein *et al* first isolated and recognized the multilineage differentiation ability of mesenchymal stromal cell [1]. The mesenchymal stromal cells can be broadly classified into two categories; MSCs derived from adult tissues such as bone marrow, adipose tissue [2] and fetal/perinatal tissues derived such as placenta [3], umbilical cord wharton’s jelly [4], amniotic membrane etc.[5]. Adult MSCs are the most commonly used MSCs but the proliferative capacity of adult MSCs are very limited, making it very difficult to scale up these adult MSCs for therapeutic applications [6]. Hence, alternate source of mesenchymal stromal cells is required for clinical application. The Mesenchymal stromal cells from extra embryonic tissues is an ideal choice for mesenchymal stem cells, as it can overcome the proliferative limitation posed by adult MSCs. Further, fetal MSCs has proliferation capacity, ease of scalability, differentiation plasticity and exhibits some of the gene expression characteristic features of embryonic stem cells without any tumorigenicity. Additionally, the immunomodulatory potential of fetal MSCs renders them as an attractive choice for regenerative medical applications [7]. In 1656 Thomas Wharton first reported the description of human umbilical chord [8]. McElreavey et al., [9] in 1991 first isolated the mesenchymal stromal cells from wharton’s jelly portion of the umbilical cord. Previous studies indicate that WJ-MSCs can be used for broad range of applications such as neurological disorders [10], kidney injury [11], lung injury [12], orthopedic injury [13], liver injury [14], cancer therapy [15]. Recent advances suggest that WJ-MSCs reinforced with microparticles [16] and scaffolds [17] can be effectively used for variety of clinical applications. Auxiliary reports suggest that paracrine factors secreted by the MSCs play a very vital role in therapeutic, immunomodulatory and tissue regeneration capabilities of MSCs [18].

Fetal bovine serum (FBS)/fetal calf serum (FCS), is routinely used culture supplement for animal cell culture applications. However, use of FBS pose the risk of xenogenic contamination leading to immunological complications during transplant applications [19]. This limitation has opened up the search to find suitable alternative supplements such as human serum [20], animal serum free synthetic substitutes [21], human platelet lysate [22] etc., for animal cell culture applications. In this study, we have standardized the protocol for isolation and characterization of human wharton’s jelly MSCs using HPL (Human Platelet Lysate) cell culture supplement. Human Bone marrow MSCs were used as a reference for comparative analysis of the mesenchymal stem cells. Further, these MSCs along with the combination of decellularized amniotic membrane was used to test the wound healing properties by creating skin injury in SCID mice models. Biomechanical properties of regenerated skin along with traditional histopathological staining techniques (Messon’s trichrome staining) were used to characterize the wound healing potential of WJ-MSC. Finally, the fate of the transplanted cells was determined by ICG labeling, which is relatively unknown after *in vivo* injections. Conventional techniques employ luciferase-based method for cell tracking which involves compromising the integrity of the cellular genome because of integrating viral vectors. In 1995, kodak research laboratories developed Indocyanine green (ICG), a cyanine dye for near infrared imaging. Subsequently, USFDA has approved ICG for variety of diagnostic applications at a clinical level [23]. In this study, we have successfully employed ICG based cell labeling technique for real-time cell tracking in SCID mice on IVIS live cell imaging system.

## Materials and Methods

### Collection of Human Umbilical Cord and Placenta Samples

Human umbilical cord and placenta (biological waste material following delivery) was collected after obtaining written consent from the patients undergoing full-term pregnancy elective caesarean. We had written consent obtained from each participant donors. The Study was carried out after Institutional Review Board (IRB) approval from Christian Medical College, Vellore, India.

### Animals

Black SCID Mice (B6.CB17-prkdc^Scid^/SzJ) was used in this study. The mice were purchased from Jackson Laboratory (Bar Harbor, ME, USA). Institutional animal ethics committee approved the experiments. The study was carried in accordance with the institutional guidelines for animal care of Christian Medical College, Vellore, India.

### Isolation of Human Wharton’s Jelly MSCs (WJ-MSCs)

Human umbilical cord was obtained from the term placental samples. Isolation of the Wharton’s Jelly MSCs was carried as previously described by Alp Can and Deniz Balci [24] with some modifications. The umbilical cord was cut into 5 cm pieces. The tissue sample was washed with Dulbecco’s Phosphate Buffer Saline (DPBS) thoroughly. The cord was cut along the horizontal axis and the vein and arteries were removed. The remaining tissue sample was minced mechanically. The minced was washed twice DPBS and subjected to sequential digestion with collagenase I, and dispase respectively. The tissue was incubated with 12.5 U/mL collagenase I for 12 hours at 37°C in shaking water bath. After incubation, dispase (2% w/v) was added into the same mixture and sample was incubated at 37°C for additional 2 hours. The enzyme digested tissue samples were passed through 250 µm metal sieve. The retained material was washed with DPBS and subjected to RBC lysis. Later, the tissue samples were thoroughly washed DPBS containing 1% penicillin/streptomycin and 2.5 µg/mL amphotericin B. The sterilized tissue samples were placed in T175 cm^2^ flask containing alpha minimum essential media (αMEM) supplemented with 1 mM L-glutamine, 5% human platelet lysate (HPL) and 1% penicillin/streptomycin. On every 3^rd^ day half of the old culture media was replaced by fresh media. Mesenchymal stromal cell colonies were visible by 7 days. By the end of 2^nd^ week the confluent flask was subjected to passaging by 0.05% trypsin. Further passaging of the cells was carried out using T75 cm^2^ flask once in three days.

### Human Platelet Lysate

Human platelet lysate was prepared as described by Patrick Horn et al., [19]. Platelet units were obtained from hospital blood bank (Christian Medical College, Vellore), pooled together and distributed in the aliquots of 45 mL. The aliquots were initially frozen at −80°C for overnight and thawed at 37°C this cycle was repeated twice in order for lysis to occur. To avoid gelatinization of the lysate 2 U/mL heparin was added. The membrane fragments were removed by subjecting the lysate to 2600 g for 30 minutes and the supernatant was passed through 0.2 µm PVDF membrane filters (Millipore, Billerica, MA, USA). The HPL was aliquoted and stored at −80°C until further use.

### Isolation and Characterization of Human Placental MSCs

Isolation and characterization of human placental MSCs was carried out as described by Sabapathy et al., [3].

### Antibodies

Refer to **Table S2 in File S1** for information on primary and secondary antibodies used for flow cytometry and immunostaining.

### Flow Cytometry Analysis

For flow cytometry analysis, the MSCs after trypsinization was distributed in the aliquots 10^5^ cells per antibody analysis. For control, unstained cells and IgG isotype antibody was used. The cells were stained with antibodies for 20 min at room temperature. FACS Calibur (Becton Dickinson, USA) instrument and Cell Quest (Becton Dickinson, USA) software was used for acquiring and analysis of the samples. For analysis, minimum of 10^4^ gated events was recorded [3].

### *In vitro* Multilineage Differentiation Analysis

Differentiation analysis was carried out to demonstrate the ability of the human WJ-MSCs into all three germ layers under *in vitro* conditions.

### Adipocyte Differentiation

Adipocyte differentiation Media (Invitrogen, CA, USA) was used for differentiation of hWJMSCs. The differentiation was carried out as mentioned in the manufactures protocol. About 5×10^4^ cells were used for differentiation in a 24 well plate. The media was changed alternate days for about 30 days. The differentiation was confirmed using Oil Red O staining. Leica inverted microscope was used for imaging (Leica, Solms, Germany) [3].

### Chondrocyte Differentiation

For chondrocyte differentiation, 1×10^6^ million cells were subjected to centrifugation at 300 g. The cell pellet was treated with chondrocyte differentiation media (Invitrogen, CA, USA) for 30 days. The differentiation was confirmed by Saffranin O staining [3].

### Osteoblast Differentiation

For Osteoblast differentiation, 5×10^4^ cells were seeded in a well of a 24 well plate. The differentiation was carried out with the help of osteocyte differentiation media (Invitrogen, CA, USA) for 30 days [3]. The Differentiation was confirmed by VonKossa staining and alkaline phosphatase staining.

### Neural Differentiation

The neural differentiation of WJMSCs was carried out using β-mercaptoethanol as described by Woodbury et al., [25]. Neural differentiation was confirmed using Neuroglia2 (NG2) by immunostaining.

### Smooth Muscle Cell Differentiation

The placental MSCs have the ability to spontaneously differentiate into smooth muscle cells. The presence of vascular smooth muscles was confirmed by α-SMA staining.

### Pancreatic β Cell Differentiation

A combination of 10 mM Nicotinamide and 1 mM β-mercaptoethanol was used for differentiation of WJMSCs into Pancreatic progenitor cells [3]. Insulin and PDX1 expression levels were confirmed by immunostaining.

### Retinal Progenitor Cell Differentiation

The differentiation of WJMSCs into photoreceptor cells was carried out using media containing 50 µM taurine and 1 mM β-mercaptoethanol for 8 days. The media was changed alternate days. The differentiation of the cells into photoreceptor cells was confirmed by rhodopsin staining [3].

### Cell Cycle Analysis

For cell cycle analysis, 10^6^ cells were fixed with ice-cold methanol by intermittent vortexing. Prior to analysis by flow cytometer, the cells were treated with RNaseA 10 µg/mL and stained with 50 µg/mL propidium iodide [3].

### Apoptosis Analysis

Apoptosis analysis of the WJMSCs was carried out using Annexin V kit (BD Pharmingen, CA, USA). The cells were stained with Annexin V and 7-AAD before acquiring and analysis the cells through flow cytometry.

### Redox Potential Analysis

For redox potential analysis, 10^5^ cells were first seeded in T25 flask. Once the flask reached the confluency of 70–80%, the cells were trypsinised and fresh culture media containing 10 µM DCFDA was added and incubated at 37°C for 30 minutes. For positive control, 50 µM H_2_O_2_ was added along with the dye. The cells were analyzed by flow cytometry.

### Soft Agar Assay

Soft Agar Assay was carried out as described by Sabapathy et al., [3].

### Isolation of Total RNA and cDNA Synthesis

The Isolation of total RNA was carried out using Trizol (Invitrogen, CA, USA). Superscript III first-strand synthesis system (Invitrogen, CA, USA) was used to prepare the cDNA using isolated total RNA.

### Quantitative Real Time PCR Analysis

The real time PCR analysis of the sample was carried out with the help F-410 DyNAmo HS SYBR green master mix (Thermo Scientific, MA, USA) using QuantStudio 12 K flex real-time PCR system (Invitrogen, CA, USA). Beta actin was used for normalizing the gene expression levels. Primer sequences used to quantify endogenous gene expression levels are listed in **Table S1 in File S1**.

### Isolation and Decellularization of Amniotic Membrane

Amniotic membrane was isolated from the placenta samples. Amniotic membrane was washed with PBS thoroughly before proceeding towards decellularization. The decellularization of the amniotic membrane was carried out as described previously by Stacy Paul et al., [26]. Briefly, the membrane was initially incubated with hypotonic 10 mM Tris buffer containing 0.1% EDTA and 10 kiu/mL aprotonin for overnight. Further, the tissue was treated with 0.03% SDS, 0.1% EDTA and 10 kiu/mL aprotonin for 24 hours. Finally, the membrane was washed with PBS thoroughly and immersed in reaction buffer containing 50 mM Tris-HCl, 50 U/mL DNase I, 1 U/mL RNase, 10 mM Magnesium Chloride, 50 µg/mL bovine serum albumin for 3 hours. The sterilization of the scaffold was carried out sequentially by washing the scaffold with 0.1% peracetic acid, 70% ethanol and PBS solution containing 1% penicillin/streptomycin, and 2.5 µg/mL amphotericin B.

### Histopathology

The umbilical cord tissue was fixed with 10% formalin for 24 hours. The tissue was paraffin embedded and sectioned vertically along the axis. The section was deparaffinized and subjected to masson’s trichrome staining.

### Karyotyping

Human WJMSCs was subjected to karyotyping cytogenetic analysis to check the integrity of the chromosome. The standard procedure was performed at 400–550 GTG band level using metaphase chromosomal preparation. Zeiss axioplan microscope (Carl Zeiss, Oberkochen, Germany) was used for analyzing the chromosome.

### Skin Injury SCID Mice Model

Black SCID mice (B6.CB17-prkdc^Scid^/SzJ) were used to study the skin injury model. The mice were initially subjected to general anesthesia by administering 50 mg/mL ketamine–6 mg/ml xylazine mixture intra-muscularly. The skin of the mice was first shaved and hair removal cream was applied to make the area very smooth. An area of 1 cm^2^ dorsal skin was cut open to create a full skin excision wound. About 1×10^6^ human WJMSCs in 100 µl PBS were injected into the skin on all the 4 direction (n = 3). In another experiment, 1×10^6^ cells were seeded onto the amniotic membrane scaffold and the scaffold was sutured onto the surface of the wound (n = 3). In control mice neither cell transplantation nor membrane grafting was carried out (n = 3). All the experiments were repeated thrice to rule out the variabilities. After 14 days, the surface wound area was subjected to histopathology and tensile strength analysis.

### Biomechanical Strength and Other Parameter Assessment of Regenerated Skin Tissues

Tensile testing machine (Tinius Olsen, HK5S model) of 5 KN load forming cell was used to characterize the mechanical properties of the regenerated tissues. Tensile testing of the tissues was carried out at room temperature at a stretch rate of 1 mm/min until failure. The stress, strain, young’s modulus, stiffness, modulus of toughness and ultimate tensile strength of the material was calculated from the force and extension values obtained from the experiment.

### Optimal Indocyanine Green (ICG) Concentration for Labeling and *In vivo* Tracking of WJ-MSCs

The 5×10^4^ WJMSCs were seeded per well of a 24 well plate in 6 wells. When the cells reached the confluency of 80%, the cells were treated with varying concentration (0.1 mg/mL–2 mg/mL) for 30 minutes at 37°C. The cells were washed twice with DPBS and cultured in complete αMEM media. Untreated cells were used as control. The total flux emitted from each well was measured using IVIS imaging system (PerkinEmler, MA, USA).

### Transplantation of ICG Labelled WJ-MSCs

For transplantation, 1×10^6^ cells was resuspended in PBS solution and labeled with 0.2 mg/mL ICG for 30 minutes at 37°C. After incubation, the cells were washed with PBS twice and the cells were resuspended in 100 µl of PBS before transplantation.

### *In vivo* near Infrared Fluorescence Imaging in IVIS System

The *in vivo* imaging of the transplanted cells was carried out using IVIS imaging station (PerkinEmler, MA, USA). The animals were anesthetized with the help of isoflurane anesthesia system.

### Statistical Analysis

Analysis of variance test was carried out for statistical comparisons between multiple groups by using Sigma Plot V11.0 software. The results are expressed as mean ± standard deviation. The results with *P<0.05 was interpreted to be significant.

## Results

### Human Wharton’s Jelly-derived MSCs (WJ-MSC) Maintains Phenotypic Attributes and *In vitro* Differentiation Plasticity during Long-term Culture

**Figure 1:**
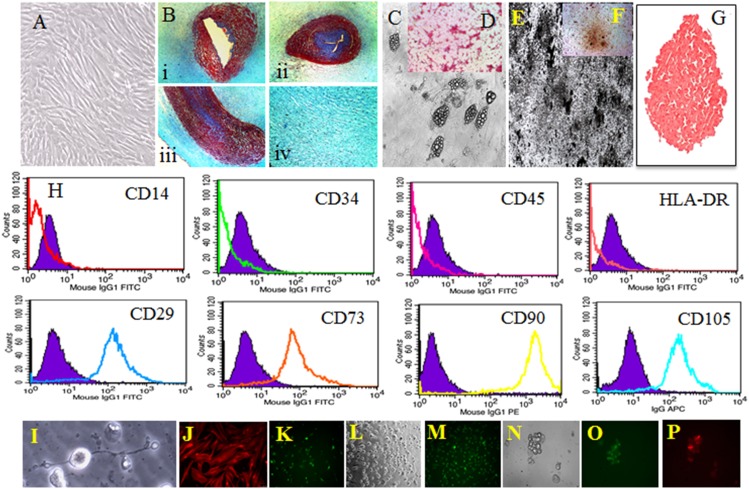
Isolation and primary characterization of WJ-MSCs. (A) WJ-MSCs Passage 5 in HPL; (B) WJ-MSCs masson’s trichrome staining; (C) Adipocyte differentiation of WJ-MSCs; (D) Oil Red O Staining; Osteocyte differentiation, Von kossa Staining (E) and Alkaline Phosphatase staining (F); (G) Chondrocyte Differentiation, Safranin O Staining; (H) Flow cytometry Analysis of WJ-MSCs; (I) Neural Differentiation, Neuroglia2 immunostaining (J); (K) Smooth muscle actin staining; (L) Photoreceptor cell differentiation, Rhodopsin staining (M); (N) Pancreatic Progenitor differentiation, (O) Insulin, (P) PDX1.

Umbilical cord is the connective tissue that connects fetus and the human placenta. Umbilical cord consists of two umbilical arteries and one umbilical vein surrounded by wharton’s jelly, gelatinous proteoglycan rich matrix (Fig. 1B). Amniotic epithelium forms the outer layer of the umbilical cord [27]. MSCs were isolated from wharton’s jelly region of human umbilical cord (Fig. 1A). These MSCs were split into 1∶3 ratios with the help of trypsin. Passaging of the cells was carried out once in 2 days and was not allowed to be more than 60–70% confluent. These MSCs could be easily expanded up to 25 passages without drastic changes in their morphology and other intrinsic cell characteristics. In 2006 International Society for Cellular Therapy (ISCT) laid the minimum criteria for the cells to be labeled as MSCs [28]. The human wharton’s jelly MSCs isolated from umbilical cord successfully meets all the criteria mentioned by the society. The MSCs were capable of differentiating into adipocytes (Fig. 1C), osteocytes (Fig. 1E) and chondrocytes (Fig. 1G). These multipotency property of the MSCs were confirmed by oil red O staining (Fig. 1D), von kossa staining (Fig. 1E), alkaline phosphatase staining (Fig. 1F) and saffranin O (Fig. 1G) staining respectively. The flow cytometry data (Fig. 1H) indicated that the cells stained highly positive for CD29 (Integrin β1), CD73 (Ecto-5′-nucleotidase), CD90 (Thy 1) and CD105 (Endoglin). There was no expression of CD14, CD34, CD45 (PTPRC) and HLA-DR markers in the isolated MSCs. The immune staining data indicate that the MSCs stained positive for smooth muscle actin, which is the marker for smooth muscles (Fig. 1K). Apart from the multipotency property, the pluripotency property of the MSCs was confirmed by differentiating the MSCs into ectoderm and endoderm lineages. The MSCs were differentiated into neural (Fig. 1I) and photoreceptor cells (Fig. 1L) for ectoderm differentiation. The pancreatic progenitor cell differentiation was carried out to confirmed the endoderm differentiation capabilities of the MSCs. Neuroglial2 (Fig. 1J) and rhodopsin (Fig. 1M) immunostaining was carried out to confirm neural and photoreceptor cell differentiation. The Pancreatic progenitors were stained with PDX1 (Fig. 1P) and insulin (Fig. 1O) for confirmation.

### Human WJMSCs Retains Many Intrinsic MSC Characteristics during Long-term *In vitro* Cultures

**Figure 2:**
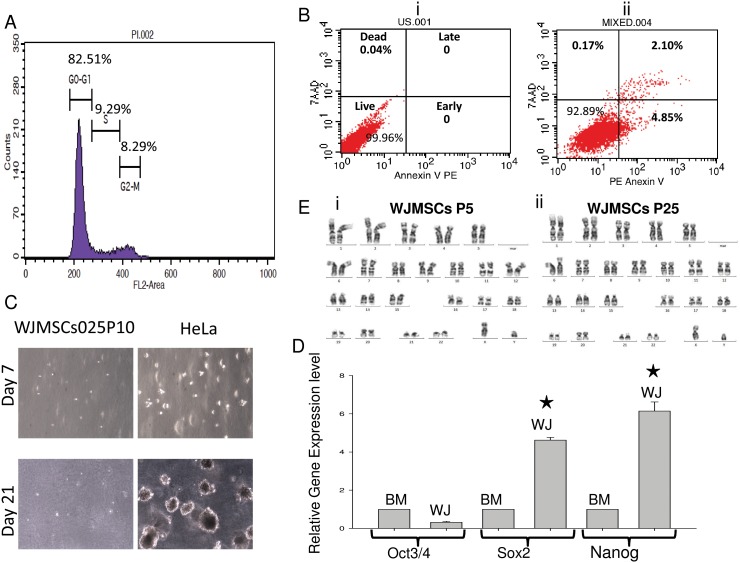
Secondary Characterization of WJ-MSCs. (A) Cell cycle analysis, passage 10; (B) Apoptosis analysis, passage 10; (C) Soft agar assay between WJ-MSCs and HeLa cells; (D) Real time analysis of endogenous pluripotency marker expression level; (E) Cytogenetic analysis of human WJMSCs.

Apart from the primary flow cytometry and differentiation analysis, the cells were subjected to additional secondary characterization studies such as cell cycle, apoptosis, soft agar assay, pluripotency marker analysis by QPCR and cytogenetic analysis in order to establish the safety of the isolated and *in vitro* expanded MSCs. The cell cycle profile of the MSCs carried out by flow cytometry analysis was normal (Fig. 2A) during the each alternate passaging of cells compared with early passage (P0). Extensive passaging of the MSCs did not exhibit any aberrant cell cycle profile. The health of the cells was confirmed by apoptosis analysis (Fig. 2B). The apoptosis analysis indicates that about 95% of the human WJMSCs displayed healthy phenotype. The results suggest that even at the later passages over 90% of the cells were healthy. To study the *in vitro* tumorigenesis property of WJMSCs, the MSCs were subjected to soft agar assay (Fig. 2C). The HeLa cells were used as a control for tumorigenesis assay. At the end of 21 days WJ-MSCs did not exhibit any spheroid formation. However, HeLa cells demonstrated tumor sphere forming capability as early as seven days. These spheres had grown larger in size by the end of 21 days. Even WJ-MSCs at passage 30 did not form spheroids. Suggesting that even after repeated passaging the cells did not acquired tumor formation capabilities. QPCR analysis of WJ-MSCs carried out to compare the endogenous pluripotency marker expression of WJ-MSCs with that of adult bone marrow MSCs (Fig. 2D). The results suggest that the endogenous pluripotency marker, *Nanog* and *SOX2* levels were significantly higher in fetal WJ-MSCs compared to adult bone marrow derived MSCs. Further, to check the chromosomal abnormality the cells were subjected to karyotyping analysis. The results from the karyotyping analysis as represented in figure 2E indicate that there is no chromosomal abnormality in the isolated and long-term culture expanded WJ-MSCs. Even at higher passage, passage 25 the chromosomal integrity of the cells remained intact.

### Perinatal MSCs Expresses Higher Levels of Immunomodulatory Compounds Compared to Human Bone Marrow-derived MSC (BM-MSC) in Presence of Pro-inflammatory Cytokines

**Figure 3:**
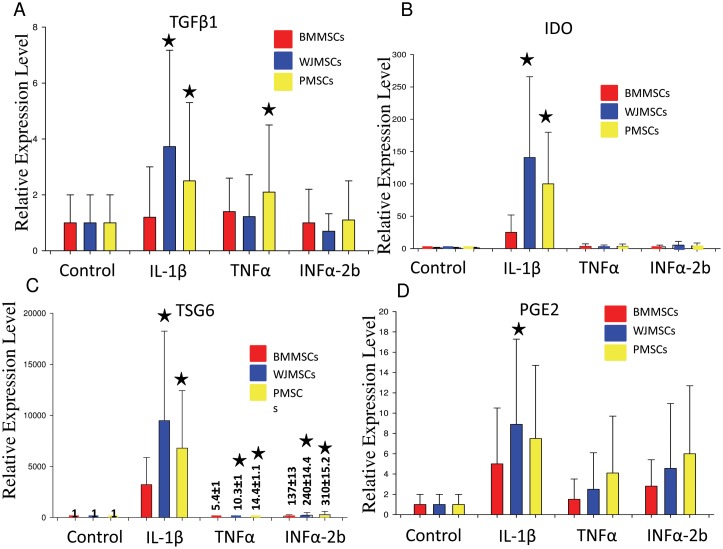
Immunological Characterization of WJ-MSCs. (A) TGFβ1, (B) IDO, (C) TSG6, and (D) PGE2 expression from Bone marrow, Wharton’s Jelly and Placental stromal cells in presence of IL-1β, TNFα, INFα-24 for 48 hours.

Earlier studies have indicated that MSCs play a very effective role in immunomodulation. In this study the extent of immunomodulation between human adult bone marrow derived MSCs were compared with human extra embryonic tissue derived WJ-MSCs and placenta-derived MSCs. The MSCs were exposed to IL-1β (10 ng/ml), TNFα (10 ng/ml) and INFα-2b (150 U/ml) containing media for 48 hrs. The relative gene expressions of immunomodulatory factors such as TGFβ1, IDO, TSG6 and PGE2 was estimated by quantitative real-time PCR (Fig. 3). The normalization of the expression levels was carried out with the help of house-keeping gene β-actin expression levels. Overall results suggest that fetal MSCs significantly expressed highly level of immunomodulatory factors when compared to adult bone marrow derived MSCs.

### WJ-MSCs Seeded on Decellularised Amniotic Membrane Augments Scar-free Wound Healing with Hair Growth

**Figure 4:**
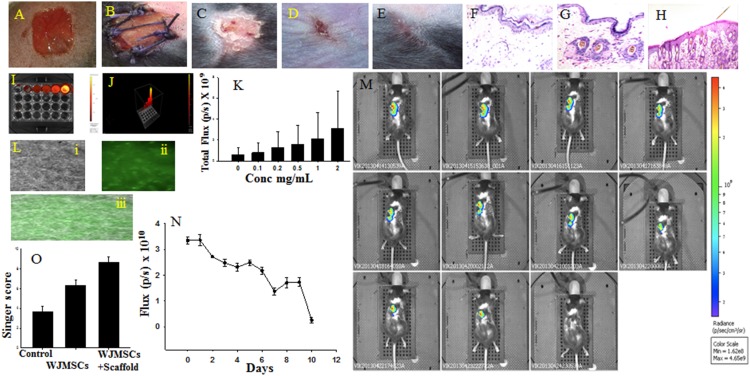
Mice skin injury model and *in vivo* tracking of cells. (A) Dermal injury; (B) Amniotic membrane containing WJ-MSCs grafting; Area of injury after 14 days, (C) Control, (D) WJ-MSCs injection, (E) Amniotic membrane + WJ-MSCs; H&E staining, (F) Control, (G) WJ-MSCs injection, (H) Amniotic membrane + WJ-MSCs; *in vitro* analysis of ICG, (I) 2D, (J) 3D; (K) Total flux of ICG in different concentration; (L) GFP labeled WJ-MSCs seeded on amniotic membrane, (i) Phase contrast, (ii) fluorescent image and (iii) merged image. (M) IVIS image of amniotic membrane containing ICG labeled WJ-MSCs grafted mice showing fluorescence for 11 days, (N) decrease in total flux of ICG with passage of time; (O) Singer score of regenerated tissue.

SCID mice were employed to study the skin wound healing. The mice divided into three groups based on the type of treatment. In the first group, only WJ-MSCs was injected, the second group consisted of grafting of WJ-MSCs seeded decellularized amniotic membrane over the wound site (Fig. 4B), and in the control group only PBS was injected. Natural decellularised amniotic membrane was used as a scaffold for culturing WJ-MSCs (Fig. 4L) before transplantation. After 14 days, the progress of wound healing was interpreted by subjecting the regenerated skin tissue, which replaced the scarred tissue (Fig. 4C–E) by histopathological examination (Fig. 4F–H; Figure S1 in File S1). The wound healing score was carried out in accordance with singer’s classification (Figure S1 in File S1) [29]. The singer score points that combinatorial therapy of WJMSCs and decellularized amniotic membrane scaffold showed the best regeneration (Fig. 4O; Figure S1 in File S1). The final aim of the study was to track the transplanted cells successfully *in vivo*. ICG (Indocyanine Green) was used to label the cells for tracking them under *in vivo* condition. To optimize the labeling concentration, the cells were initially labeled with different concentrations of ICG dye. The concentrations between 0.1–2 mg/mL were used for labeling the cells under *in vitro* conditions. The total flux emitted was measured using IVIS imaging system. The concentrations of ICG and total flux (p/s) were plotted. The results indicate that the intensity of the flux is directly proportional to concentration of ICG. Further, previous studies suggest that the concentration greater than 0.5 mg/ml is cytotoxic to the cells [30], [31], [32]. Hence, 0.2 mg/ml ICG dye was selected for effective labeling and *in vivo* tracking of the cells. Initially, the cells were labeled with ICG for one hour at 37°C. The labeled cells were seeded on the decellularized amniotic membrane for 24 hours at 37°C and then transplanted onto the injured mice. The photon emission was measured using IVIS imaging system daily till the intensity levels diminished. The results show that the labeled WJMSCs were tracked successfully *in vivo* for around 10 days.

### Regenerated Skin Tissue from SCID Mice Transplanted with WJ-MSC Seeded on Decellularized Amniotic Membrane Demonstrated Best Biomechanical Properties

**Figure 5:**
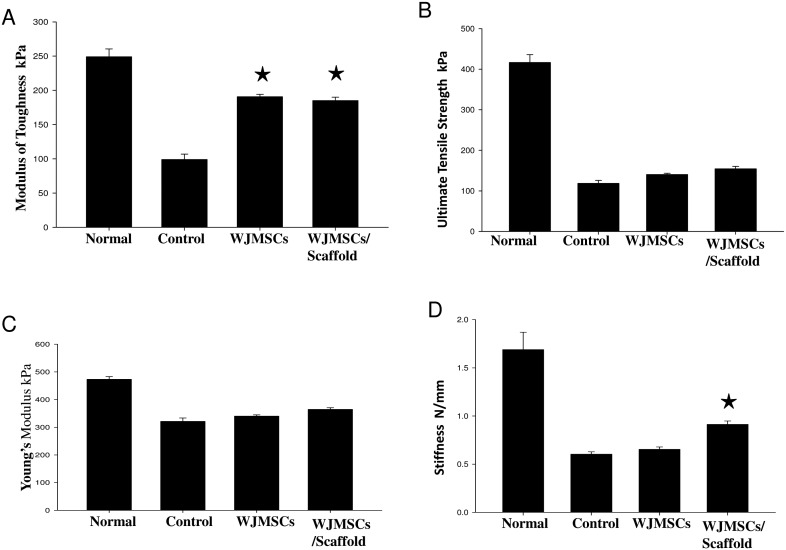
Mechanical Characterization Day14 Post Injury. (A) Modulus of Toughness; (B) Ultimate tensile strength; (C) Young’s Modulus; (D) Stiffness.

Apart from the regular histopathological evaluation of regenerated tissue we wanted to understand the biomechanical properties of the regenerated skin tissue. The skin tissues to be used for biomechanical characterization (Fig. 5) was classified into 4 groups, normal, control (PBS Injected), WJMSCs injected and combination of WJMSCs/Scaffold based on the mode of treatment. The tissues were subjected to tensile testing machine for recording force and extension primary parameters. From the primary parameters, secondary parameters such as stress, strain, modulus of toughness (Fig. 5A), ultimate tensile strength (Fig. 5B), young’s modulus (Fig. 5C) and stiffness (Fig. 5D) was calculated. Analysis of the all biomechanical parameters demonstrates that biomechanical property of WJMSCSs/Scaffold was better compared to all other groups including WJMSCs injected tissue.

## Discussion

Use of FBS as a media supplement has lot of disadvantages for clinical applications. Use of platelet lysate as an alternative *in vitro* cell culture supplement might lower the limitations, posed by the use of fetal bovine serum (FBS). In this study the human WJ-MSCs was successfully isolated and *in vitro* culture expanded by using 5% HPL supplement in α-MEM Media. The calculated population doubling time was around 10 hours. The isolated human WJ-MSCs were subjected to primary characterization analysis such as flow cytometry and differentiation analysis. The characteristics property exhibited by the WJ-MSCs were similar to that of adult bone marrow derived MSCs. Previous studies have indicated that human WJ-MSCs exhibits similar phenotypic characteristics, surface marker expression, differentiation ability, immunomodulatory effects and paracrine factors similar to that of adult bone marrow MSCs [33]. Nonetheless, Fetal WJ-MSCs exhibited high proliferation capacity compared to adult BM-MSCs thus can be scaled up easily for regenerative medical applications. WJ-MSCs exhibited the capacity to transgress across the trans-lineage barrier and able to efficiently differentiate into ectoderm (neural cells), mesoderm (adipocyte, osteocyte, chondrocyte, smooth muscles etc.) and endoderm (Pancreatic progenitors). Additionally, isolated human WJ-MSCs were subjected to secondary characterization studies such as cell cycle analysis, apoptosis analysis, cytogenetics and *in vitro* tumorigenesis analysis. The overall results suggest that isolated cells were very healthy and did not form any aberrant cell types upon extended passaging. The MSCs isolated from extra-embryonic tissues such as umbilical cord has pluripotency marker expression level some were midway between embryonic and adult cells [6]. Previous studies have indicated the presence of higher expression of pluripotency marker such as Nanog, OCT4, Tra-1-81 etc., in the cells isolated from the extra embryonic tissues [34]. According to our data, comparative real-time PCR analysis between the adult bone marrow derived MSCs and WJ-MSCs indicate the presence of considerable levels of Nanog and Sox2 expression. The presence of high level of pluripotency marker in the fetal MSCs compared to adult BM-MSCs might play a vital role in improved proliferation and differentiation capabilities. Initial reports on immunoregulatory property of adult MSCs were demonstrated just around a decade back [35], [36]. MSCs have the ability to modulate both innate and adaptive immune response by complex interactions with T cells, B cells, NK cells, dendritic cells, macrophages, neutrophils, toll like receptors etc., [37]. When the MSCs were subjected to proinflammatory cytokines such as IL-1β, TNFα and INFα-2b, there was a differential expression level of immunomodulatory molecules such as TGFβ1, IDO, PGE2 and TSG6. Over fetal WJMSCs exhibited comparatively elevated expression of immunomodulatory molecules when compared to adult bone marrow MSCs. The WJ-MSCs express lower levels of HLA-I thus provokes no immune response from the host. Our data is consistent with previously reported result that, WJMSCs exhibits better immunomodulatory properties compared to adult BM derived MSCs [38]. Nonetheless, still the complex mechanism of immunomodulation of MSCs remains shrouded in mystery. Wound healing is a convoluted process involving coordinated interplay between cells, growth factors and extracellular proteins. The MSCs plays a vital role by secreting important paracrine factors that helps coordinating the wound healing process. The prior reports on various animal models and clinical trials indicate MSCs play a beneficial role in augmenting the wound healing process [39]. In this study, the data suggest that the grafting of the WJ-MSCs seeded decellularized amniotic membrane exhibited better wound healing capabilities compared to injection of MSCs alone. The histological study of regenerated wound tissue two week post injury suggest that glandular tissues were better developed and organized in membrane grafted tissue compared to control or plain WJ-MSCs injected animal model. Further, one of our main goals was to successfully track the transplanted cell under *in vivo* conditions. ICG is FDA approved dye with an absorbance and emission peak at 780 nm and 830 nm respectively [40]. It is approved for clinical applications such as cardiac perfusion etc. In this study, we have optimized the protocol for efficient labeling of the cells. The ICG concentration of 0.2 mg/ml was successfully employed for both cell labeling as well as *in vivo* imaging. Moreover, previous reports have indicated that ICG greater than 0.5 mg/ml might decreases the viability of the cells. The ICG labeled cells seeded on decellularized amniotic membrane was grafted onto the area of dermal injury. The labeled cells were tracked via IVIS imaging system for almost 10 days. Thus, non-toxic ICG labeling can be successfully extrapolated for clinical applications to track the transplanted cells. Mechanical characterization data of the regenerated tissue supplemented our finding that combination of WJ-MSCs/scaffold is suitable for wound healing. The interpretation of modulus of toughness, ultimate tensile strength, young’s modulus and stiffness provides sufficient evidence that the wound healing capabilities of WJMSCs/Scaffold was significantly better compared to control. By taking account of only the mechanical parameters, there was no significant difference between WJ-MSCs and WJ-MSCs/Scaffold transplanted groups. However, Ultimate tensile strength, young’s modulus and stiffness values of WJ-MSCs/Scaffold transplanted group were more compared to only WJ-MSCs injected group.

## Conclusions

Human platelet lysate was used to successfully expand the isolated WJ-MSCs from human wharton jelly tissue of umbilical cord. The primary and secondary characterization of isolated WJ-MSCs was carried to ascertain the phenotypic as well as genotypic features of the MSCs. The fetal MSCs exhibited greater stemness compared to gold standard adult bone marrow derived MSCs. Moreover, the higher immunomodulatory factors were secreted by the extra embryonic tissue derived MSCs compared to adult MSCs under proinflammatory conditions. SCID mice skin injury model suggest that combination of natural decellularised amniotic membrane scaffold along with WJMSCs can be effectively used to treat the skin injury. Lastly, the data indicate that ICG can be used as a reliable source for tracking of the cells during *in vivo* applications.

## Supporting Information

File S1 **Supporting figure and tables. Figure S1**, Histopathological evaluations of regenerated tissue using singer classification for quantifying cutaneous wounds (Masson Trichrome Staining). **Table S1**, List of Q-PCR primers used in this study. **Table S2**, List of antibodies used in this study.
(DOCX)
