# Supplementary material for: Human Wharton’s Jelly Mesenchymal Stem Cells Plasticity Augments Scar-Free Skin Wound Healing with Hair Growth
Source: PLoS One. 2014 Apr 15;9(4):e93726. doi: 10.1371/journal.pone.0093726 (PMC3988008; doi:10.1371/journal.pone.0093726)
Supplement: File S1 — Supporting figure and tables. Figure S1, Histopathological evaluations of regenerated tissue using singer classification for quantifying cutaneous wounds (Masson Trichrome Staining). Table S1, List of Q-PCR primers used in this study. Table S2, List of antibodies used in this study. (DOCX) [file pone.0093726.s001.docx]

**Supplementary DATA**

**Supplementary Figure S1:** Histopathological evaluations of regenerated tissue using singer classification for quantifying cutaneous wounds (Masson Trichrome Staining).

.


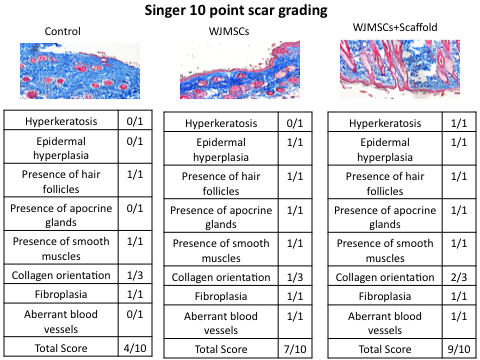


**Table S1: QPCR Primers**

| **Primers** | **Sequence 5'-3'** | **Reference** |
| --- | --- | --- |
| Beta Actin Fwd | CCT TCC TGG GCA TGG AGT CCT | [3] |
| Beta Actin Rev | GGA GCA ATG ATC TTG ATC TTC |  |
| Endo OCT4 Fwd | CCT CAC TTC ACT GCA CTG TA |  |
| Endo OCt4 Rev | CAG GTT TTC TTT CCC TAG CT |  |
| Endo SOX2 Fwd | CCC AGC AGA CTT CAC ATG T |  |
| Endo SOX2 Rev | CCT CCC ATT TCC CTC GTT TT |  |
| Endo Nanog Fwd | GCT TGC CTT GCT TTG AAG CA |  |
| Endo Nanog Rev | TTC TTG ACT GGG ACC TTG TC |  |
| Human IDO Fwd | CAAAGGTCATGGAGATGTCC | [41] |
| Human IDO Rev | CCACCAATAGAGAGACCAGG |  |
| Human PGE2 Fwd | GAC CGC TTA CCT GCA GCT GTA C | [42] |
| Human PGE2 Rev | TGA AGT TGC AGG CGA GCA |  |
| Human TGFβ1 Fwd | CCCAGCATCTGCAAAGCTC | [43] |
| Human TGFβ1 Rev | GTCAATGTACAGCTGCCGCA |  |
| Human TSG6 Fwd | GGCCATCTCGCAACTTACA | [44] |
| Human TSG6 Rev | CAGCACAGACATGAAATCCAA |  |

**Table S2: Antibodies**

| **Antibodies** | **Conjugate** | **Cat No** | **Company** |
| --- | --- | --- | --- |
| Anti-Human CD 14 | FITC | 11-0149 | eBioscience |
| Anti-Human CD 34 | FITC | 11-0349 | eBioscience |
| Anti-Human CD 45 | FITC | 11-9459 | eBioscience |
| Anti-Human CD 29 | FITC | 11-0299 | eBioscience |
| Anti-Human CD 73 | FITC | 11-0739 | eBioscience |
| Anti-Human CD 90 | PE | 12-0909 | eBioscience |
| Anti-Human CD 105 | APC | 17-1057 | eBioscience |
| Mouse IgG1 K isotype control | FITC | 11-4714 | eBioscience |
| Mouse IgG1 K isotype control | PE | 12-4714 | eBioscience |
| Mouse IgG1 K isotype control | APC | 17-4714 | eBioscience |
| HLA DR | FITC | 11-9956-73 | eBioscience |
| NG2 | Alexa Fluor 488 | 53-6504-80 | eBioscience |
| SMA |  | ab 54723 | abcam |
| Rhodopsin |  | ab 81702 | abcam |
| Insulin |  | 3014S | Cell Signaling |
| PDX1 | Alexa Fluor 647 | 51-6500-80 | eBioscience |

**References cited in supplementary tables:**

41. Thomas SR, Terentis AC, Cai H, Takikawa O, Levina A, et al. (2007) Post-translational regulation of human indoleamine 2,3-dioxygenase activity by nitric oxide. J Biol Chem 282: 23778–23787.

42. Sales KJ, Katz AA, Davis M, Hinz S, Soeters RP, et al. (2001) Cyclooxygenase-2 expression and prostaglandin E(2) synthesis are up-regulated in carcinomas of the cervix: a possible autocrine/paracrine regulation of neoplastic cell function via EP2/EP4 receptors. J Clin Endocrinol Metab 86: 2243–2249.

43. Xie S, Macedo P, Hew M, Nassenstein C, Lee K-Y, et al. (2009) Expression of transforming growth factor-beta (TGF-beta) in chronic idiopathic cough. Respir Res 10: 40.

44. Tan KT, Baildam AD, Juma A, Milner CM, Day AJ, et al. (2011) Hyaluronan, TSG-6, and inter-α-inhibitor in periprosthetic breast capsules: reduced levels of free hyaluronan and TSG-6 expression in contracted capsules. Aesthet Surg J 31: 47–55.
